# Grounded and embodied mathematical cognition: Promoting mathematical insight and proof using action and language

**DOI:** 10.1186/s41235-016-0040-5

**Published:** 2017-01-30

**Authors:** Mitchell J. Nathan, Candace Walkington

**Affiliations:** 1 University of Wisconsin-Madison, Educational Sciences Building, 1025 West Johnson Street, Madison, WI 53705 USA; 2 Southern Methodist University, Dallas, TX USA

**Keywords:** Educational Technology, Embodied cognition, Learning environments, Mathematics education, Proof and justification, Student learning

## Abstract

We develop a theory of grounded and embodied mathematical cognition (GEMC) that draws on action-cognition transduction for advancing understanding of how the body can support mathematical reasoning. GEMC proposes that participants’ actions serve as inputs capable of driving the cognition-action system toward associated cognitive states. This occurs through a process of transduction that promotes valuable mathematical insights by eliciting dynamic depictive gestures that enact spatio-temporal properties of mathematical entities. Our focus here is on pre-college geometry proof production. GEMC suggests that action alone can foster insight but is insufficient for valid proof production if action is not coordinated with language systems for propositionalizing general properties of objects and space. GEMC guides the design of a video game-based learning environment intended to promote students’ mathematical insights and informal proofs by eliciting dynamic gestures through in-game directed actions.

GEMC generates several hypotheses that contribute to theories of embodied cognition and to the design of science, technology, engineering, and mathematics (STEM) education interventions. Pilot study results with a prototype video game tentatively support theory-based predictions regarding the role of dynamic gestures for fostering insight and proof-with-insight, and for the role of action coupled with language to promote proof-with-insight. But the pilot yields mixed results for deriving in-game interventions intended to elicit dynamic gesture production. Although our central purpose is an explication of GEMC theory and the role of action-cognition transduction, the theory-based video game design reveals the potential of GEMC to improve STEM education, and highlights the complex challenges of connecting embodiment research to education practices and learning environment design.

## Significance

Commercially available motion-based programs for improving mathematical reasoning through action (e.g., MATHS DANCE) have long attracted the attention of educators, but with little basis in the psychological theory of how actions can reliably influence cognition. Evaluation of these programs seldom accounts for variations in learning and performance due to variation in influences such as pedagogical support (e.g., explicit prompting). Early work exploring grounded and embodied mathematical cognition (GEMC) through video game performance showed some areas of promise. As predicted, dynamic gestures reliably predicted mathematical insight, even when players were not consciously aware of the mathematical relevance of their in-game actions. GEMC also correctly predicted that proof validity would improve when players’ directed actions elicited during game play were explicitly connected to mathematical conjectures through pedagogical language. However, some findings that were at odds with our initial hypotheses reveal the gap between embodied learning theory and using theory to design effective learning environments aimed at promoting science, technology, engineering, and mathematics learning. Generally, learning theories under-constrain designs of effective instruction and learning environments, which fundamentally constricts the impact of research on practice. Engineering-based approaches of iterative design generation and refinement are needed to translate research to practice.

## Background

Principles of grounded and embodied cognition address the role of the body and body-based resources to shape cognition. Our objective is to present a theory of grounded and embodied mathematical cognition (GEMC) consistent with Stokes’ (1997) ‘use-inspired research,’ by contributing generalizable models of mathematical thinking and learning that support the application of theory to the design of effective, scalable learning experiences in science, technology, engineering, and mathematics (STEM) education. In addition to presenting the theory, we discuss how we used our theory of GEMC to guide the design of a video game that engages players’ action systems in order to promote mathematical reasoning. Our specific focus is on understanding and improving mathematical proof skills for geometry. As an example of the application of the GEMC theory, we describe early findings from a small pilot study of middle- and high-school students to understand the influences of action-based interventions on their mathematical insights and proofs. We use this occasion to discuss the inherent challenges of designing effective STEM learning environments derived from cognitive theory.

Geometric proof is a valuable content area for making strides for theories of GEMC. First, geometry is seen as the study of the properties of space and shape, and therefore should be suitable to a GEMC perspective. Second, it is an area of advanced mathematics, typically studied by students planning to attend college and other post-secondary educational programs (Pelavin & Kane, 1990). Third, geometric proof is primarily concerned with universal statements about space and objects, and therefore addresses an important area of abstract thought. Proof is an especially intriguing area of study because of the central role of conceptual understanding rather than using only ‘canned’ procedures or mathematical algorithms that might enable people to generate a valid answer with little understanding of the mathematics involved (e.g., long division). Finally, geometry plays a profound role in all of the STEM disciplines. Because of these deep connections, there is the potential for advancements in this program of research to impact mathematical reasoning and STEM education more broadly.

### Research to practice for STEM: the need to improve proof education

Justification and proof are central activities in mathematics education (National Council of Teachers of Mathematics, 2000; Yackel & Hanna, 2003). In fact, ‘proof and proving are fundamental to doing and knowing mathematics; they are the basis of mathematical understanding and essential in developing, establishing, and communicating mathematical knowledge’ (Stylianides, 2007, p. 289).

Research has long revealed that students struggle to construct viable and convincing mathematical arguments and provide valid generalizations of mathematical ideas (Dreyfus, 1999; Healy & Hoyles, 2000; Martin, McCrone, Bower, & Dindyal, 2005). Students tend to be overly reliant on examples when exploring mathematical conjectures and often conclude that a universal statement is true on the basis of only checking examples that satisfy the statement (e.g., Healy & Hoyles, 2000; Knuth, Choppin, & Bieda, 2009; Porteous, 1990). When presented with deductive proofs, students frequently find them unconvincing (Chazan, 1993), and fail to appreciate the utility of deductive reasoning for communicating generalized arguments based on logical inference (Harel & Sowder, 1998). Interviews by Coe and Ruthven (1994) showed that even advanced college mathematics students held restricted manners and attitudes toward proof. These students typically looked for standardized routines to guide their investigations, rather than seeking out methods suited to the specific conjectures at hand. Furthermore, these advanced mathematics students seldom sought out explanations that would illuminate or give them insights into the general rules and patterns, and rarely attempted to connect these patterns to broader mathematical ideas or frameworks.

In reaction, some mathematics education scholars call for more innovative approaches to proof instruction that focus on the construction and negotiation of mathematical meaning (Stylianides, 2007). Harel and Sowder (1998) define proving as ‘the process employed by an individual to remove or create doubts about the truth of an observation’ (p. 241). Thus, the process of proving encompasses a wide range of activities where students reason critically about mathematical ideas rather than focus only on an abstract, concise end product disconnected from situated reasoning.

### Grounded and embodied mathematical cognition

We view mathematical communication as a multimodal discourse practice (e.g., Edwards, 2009; Hall, Ma, & Nemirovsky, 2015; Radford, Edwards, & Arzarello, 2009; Roth, 1994; Stevens, 2012), rather than a formal, written, propositional form. When constructing valid proofs, individuals often communicate a logical and persuasive chain of reasoning using descriptive language, verbal inference, and gestures. Research on mathematicians’ proving practices has suggested that proof ‘is a richly embodied practice that involves inscribing and manipulating notations, interacting with those notations through speech and gesture, and using the body to enact the meanings of mathematical ideas’ (Marghetis, Edwards, & Núñez, 2014, p. 243). Observations show that both teachers and students use multimodal forms of talk using speech-accompanied gestures as a way to track the development of key ideas when exploring mathematical conjectures (Nathan, Walkington, Srisurichan, & Alibali, 2011). We refer to these kinds of arguments and communications as ‘informal proofs.’ While they relay the key ideas and transformations needed to explain why properties do or do not hold, they are not always organized in the propositional, deductive, and meticulous manner of formal proofs.

#### Grounded and embodied cognition

Mathematical thinking and communication, like other forms of cognitive behaviors, are of interest to the growing research program on grounded and embodied cognition (Shapiro, 2014). Grounded cognition (Barsalou, 2008, p. 619) is a broad framework that posits that intellectual behavior ‘is typically grounded in multiple ways, including simulations, situated action, and, on occasion, bodily states.’ When the focus is on the grounding role of the body, scholars typically use the more restricted term, ‘embodied cognition’. Grounded cognition is contrasted with models of cognition based on ‘AAA symbol systems’ that are abstract, amodal, and arbitrarily mapped to the concepts to which they refer (Glenberg, Gutierrez, Levin, Japuntich, & Kaschak, 2004). Yet working with symbolic notational systems and making general claims about idealized entities (such as perfect circles) through logical deduction is at the heart of mathematical proof construction. Several scholars have provided accounts of thinking about abstract entities and relationships that we never actually see or touch based on principles of grounded and embodied cognition (Casasanto & Boroditsky, 2008; Lakoff & Nunez, 2000). Thus, a central goal in this work is to explicate how a GEMC perspective accounts for seemingly abstract forms of reasoning.

#### Directed actions, gestures, and learning

One form of GEMC intervention explores the effects of directed actions on reasoning. Here we define ‘directed actions’ as physical movements that learners are instructed to formulate by some kind of pedagogical agent (Thomas & Lleras, 2009). ‘Gestures’ can be distinguished from directed actions in that they are spontaneously generated movements, often of the hand, that accompany speech and thought (Chu & Kita, 2011; Goldin-Meadow, 2005; Nathan, 2014). Our review of the literature on directed actions, gestures, and learning reveals four empirically based findings of note: (1) gesture production predicts learning and performance; (2) directed actions can influence mathematical cognition; (3) directed actions from earlier training opportunities leave a historical trace, or legacy, expressed through gestures during later performance and explanation; and (4) mathematical reasoning and learning are further enhanced when actions are coupled with task-relevant speech, leading to coordinated action-speech events that are the hallmark of contemporary gesture research. Taken together, these findings support the assertion that actions serve a valuable role in addition to language in both fostering and conveying mathematical ideas.

The first finding - that gesture production predicts learning and performance - includes content areas such as mathematics (Cook, Mitchell, & Goldin-Meadow, 2008; Valenzeno, Alibali, & Klatzky, 2003) and language (Glenberg et al., 2004; McCafferty & Stam, 2009), as well as broad influences such as general problem solving (Alibali, Spencer, Knox, & Kita, 2011; Beilock & Goldin-Meadow, 2010), inference-making (Nathan & Martinez, 2015), and cognitive development (Church & Goldin-Meadow, 1986). Conversely, when gesture production is controlled, gesture inhibition often disrupts performance and learning (Hostetter, Alibali, & Kita, 2007; Nathan & Martinez, 2015).

**Fig. 1:**
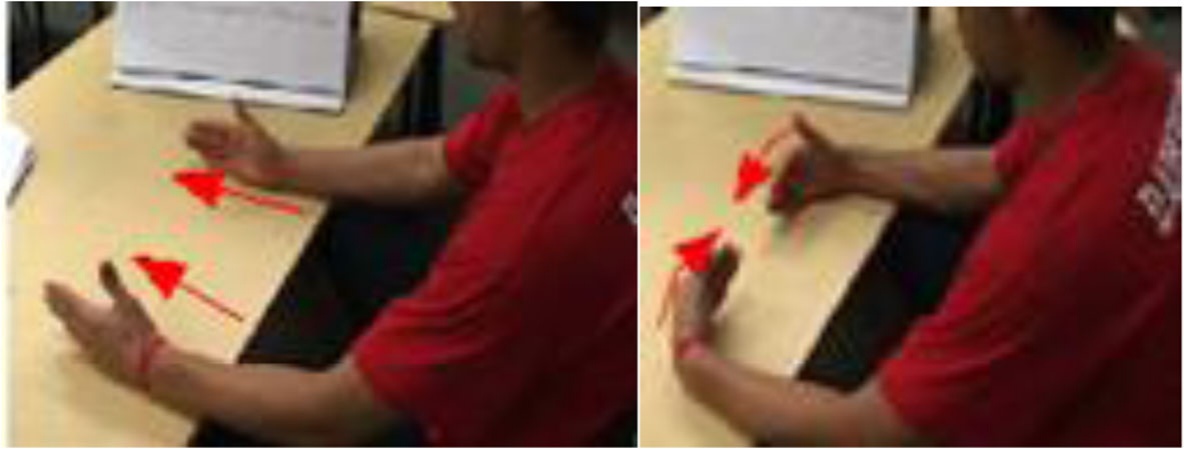
A student’s dynamic depictive gestures for the Triangle conjecture used to prove the statement that ‘The sum of the lengths of any two sides of a triangle must be greater than the length of the remaining side’

For example, the likelihood that students produced valid proofs for mathematical conjectures was positively associated with the presence of ‘dynamic depictive gestures’ (Donovan et al., 2014). Depictive gestures are gestures through which speakers directly represent objects or ideas with their bodies (e.g., forming an angle with their two hands; McNeill, 1992). Dynamic depictive gestures (which we will often refer to as ‘dynamic gestures’) are defined as those that show a motion-based transformation of a mathematical object through multiple states (Walkington et al., 2014). The odds of generating valid proofs were 4.14-times greater (95% confidence interval 1.57–10.92) for participants who produced dynamic gestures than those who did not (Donovan et al., 2014). Figure 1 shows a student making a dynamic depictive gesture while proving the statement that ‘The sum of the lengths of any two sides of a triangle must be greater than the length of the remaining side.’ The gesture uses motion-based transformations to show two sides of the triangle being unable to meet. Pier et al. (2014) demonstrated that the benefits of dynamic gestures for predicting performance on verbal mathematical proofs are over and above the effects of variations in speech.

The second finding comes from a growing body of empirical literature indicating that directed actions can influence learning. Superior problem-solving performance has been demonstrated when students follow directions to perform specific actions hypothesized to foster effective problem-solving strategies (Goldin-Meadow, Cook, & Mitchell, 2009). Thomas and Lleras (2007) showed that manipulating eye-gaze patterns can, unbeknownst to participants, affect the success of solving Dunker’s classic Radiation Problem. In mathematics, Abrahamson and Trninic (2015; Abrahamson, 2015) increased primary grade children’s awareness of mathematical proportions by engaging their hand and arm motions in order to achieve a particular goal state of the system (a green illuminated screen rather than a red one) once they enacted the appropriate (but tacit) proportions with their relative rates of dual hand movements. It may be said that their hand movements constituted a form of problem solving or epistemic action (Kirsh & Maglio, 1994). According to the authors (Abrahamson & Trninic, 2015), participants’ movements did not elicit proportional reasoning, tacitly or otherwise, because, in their view, such forms did not exist yet for these young children, who were engaged in an activity that could later give rise to the concept of proportion. Rather, students were engaged in manipulating objects in the spatial-dynamical problem space. Students could later reflect on their own emergent manipulation strategies, discern motion patterns, and then model these patterns mathematically. Thus, these physical experiences helped children’s subsequent performance symbolizing otherwise elusive multiplicative relationships. Fischer, Link, Cress, Nuerk, and Moeller (2015) used digital dance mats, Kinect sensors, and interactive whiteboards to promote physical experiences that children could use to understand the mental number line through embodied training. A mixed-reality environment developed by Lindgren (2015) fostered body ‘cueing’ that led to higher achievement and more positive attitudes toward learning by providing grounding for students’ understanding of physics principles. Enyedy and Danish (2015) also used a mixed-reality environment to support students’ understanding of Newtonian force using motion-tracking technology. They found that verbal and physical reflection on embodied activity and first-person embodied play allowed students to engage deeply with challenging concepts.

In the domain of geometry, Petrick and Martin (2012) describe an intervention where high-school students physically enacted (versus observed) dynamic geometric relations, and found that enactment improved learning gains. Shoval (2011) describes an intervention in which students made ‘mindful movements’ to kinesthetically model angles with their bodies, and demonstrated improved understanding of angles at post-test compared to a control group receiving traditional instruction. Smith, King, and Hoyte (2014) used the Kinect platform to engage students in making different types of angles with their bodies. They found that making conceptual connections between physical arm movements and the grounding metaphor ‘angles as space between sides’ allowed students to demonstrate greater understanding of estimating and drawing angles. However, they found that in order to benefit from the intervention, it was critical for students to connect their physical actions to the canonical geometric representation and to engage in dynamic movements in which they tested different hypotheses.

In our prior work, Nathan et al. (2014) showed that directing participants’ (*N* = 120) body actions affected the generation of appropriate mathematical intuition, insight, and informal proof for two different tasks. They looked at intuition and informal proof for a conjecture on properties of the lengths of sides of all triangles. They also looked at insight and informal proof for a task involving parity for a train of gears. Mathematical insights are defined as partial understandings of the key ideas underlying a mathematical system. Insight is related to but distinct from intuition (e.g., Zander, Öllinger, & Volz, 2016; Zhang, Lei, & Li, 2016): intuition draws on unconscious information to make a judgment (often Yes/No), without leaving a reportable trace of the decision-making process, whereas insights use conscious retrieval processes applied to both unconscious and conscious knowledge to report on one’s thoughts about a solution or to provide a partial solution. One of the challenges of insight processes is overcoming unhelpful associations (e.g., when conjectures about triangles inappropriately activate Pythagoras’ theorem).

Nathan et al. (2014) found that trials in which participants performed the directed actions were associated with significantly more accurate intuitive judgments on the Triangle conjecture, and more accurate insights on the Gear conjecture, than the trials that used control actions of comparable complexity that were less relevant to the mathematics. Participants who performed relevant directed actions were also significantly more likely to generate an accurate intuition on a transfer task for geometry (i.e., as extended to other polygons) than participants who performed irrelevant actions. However, participants were not more likely to have the key insight for a transfer task involving numerical parity of gears. Thus, directed actions can facilitate mathematical intuition and insight, though transfer appears to be highly task dependent.

While performing mathematically relevant directed actions facilitated key mathematical insights and intuitions for two tasks (Triangle and Gear), directed actions on their own did not lead to superior informal proofs compared to irrelevant actions. Rather, adding pedagogical language in the form of prompts (prospective statements) and hints (retrospective statements) that explicitly connected the directed actions to the tasks significantly enhanced proof performance on the Triangle task. The authors interpret the findings as raising questions about the reciprocal relations between action and cognition: actions on their own facilitate insight, while actions coupled with appropriate pedagogical language explicitly connecting the actions to the mathematical ideas foster informal proofs.

Our third finding is that actions from earlier training opportunities leave a historical trace that is evident when people later solve problems in new, related contexts. Body-based training on the Tower of Hanoi task led participants to integrate their motor experiences into their mental encoding of the objects and their subsequent solution processes (Beilock & Goldin-Meadow, 2010). Donovan et al. (2014) showed that directed actions influence later performance, and found that trained actions can leave an observable ‘legacy’ in learners’ subsequent gestures during proof production. Third and fourth graders learned to solve equivalent equations in one of two ways that involved different manual actions for the assigned condition. Participants using the two-handed ‘bucket’ strategy were significantly more likely to use both hands when solving post-test and transfer problems, and more likely to exhibit a relational understanding of the equal sign than control group participants who used no manipulatives.

In the fourth finding, we note that reasoning and learning with directed actions appears to be enhanced when the solver’s actions are coupled with task-relevant speech, such that action and language become coordinated. As noted in the proof research reviewed earlier, gestures and speech each make independent and significant contributions to predicting performance (Pier et al., 2014). Goldin-Meadow et al. (2009) performed a mediational analysis of students’ speech in their study of how directed actions influence performance on equivalent equations. Their results show that students come to apprehend the grouping strategy for solving the equivalence equations even though the strategy was depicted only through directed actions and never explicitly vocalized or gestured to the participants. Students who added grouping in their speech along with the directed actions had increased post-test performance. Their analysis showed that the action condition by itself predicted whether participants added the verbal grouping strategy to their repertoire, while verbalizing the grouping strategy more strongly predicted post-test performance. The authors propose that student-generated speech mediates learning from actions. Carrying out specific actions directs learner attention to solution-relevant features of the task, which helps students confer meaning to the actions.

Several summary points emerge from this literature: gesture production predicts learning and performance; in reciprocal fashion, directed actions are a malleable factor that may influence cognition; actions on their own tend to promote insight and intuition that is not well articulated verbally; reasoning and learning with actions is further enhanced when actions are coupled with task-relevant speech; and actions from earlier training opportunities leave a legacy trace shown in future problem solving. Together, these empirical findings form the basis for a set of hypotheses for ways to promote reasoning in STEM by exploring the mutual influences between action and cognition, as moderated by speech.

#### Action-cognition transduction

Evidence is mounting that sensorimotor activity can activate neural systems, which can in turn alter and induce cognitive states (Thomas, 2013). Recent work has also identified two critical modes of thinking: System 1 processes that are automatic, effortless, nonverbal, and largely unconscious (e.g., orienting to a sudden sound); and System 2 processes that are effortful mental activities involving agency, choice, and concentration (e.g., checking the validity of an argument; Kahneman, 2011). Whereas the influence of directed actions on cognition may largely be on automatic and unconscious System 1 processes, gestures, which are more intimately bound to language, may influence the verbal, deliberative processes of System 2 (Nathan, in press).

As reviewed above, an emerging literature on cognition and education shows that concepts can be learned through motoric (System 1) interventions. Specifically, ‘action-cognition transduction’ (ACT; Nathan, in press) explores the bidirectional relationship between cognition and action. ACT theory draws inspiration from reciprocal properties of electromechanical and biological systems relating input-output behavior. Physical devices, such as motors, acoustic speakers, light-emitting diodes (LEDs), and so forth, can run both ‘forward’ and ‘backward’; so input energy, often in the form of electric current, can be transduced when forcibly cranking the rotor of a motor (we call the ‘reverse’ motor a generator), shining a light on an LED (making a photoreceptor), or singing into a speaker (making a microphone).

Transduction behavior is evident in biological systems as well, with reported influences on cognitive processes. Niedenthal (2007) illustrates how affective state is surreptitiously induced through manipulations in the facial muscles to form specific facial expressions, which in turn influence the cognitive processing of emotion information when presented in writing, speaking, and images. Havas, Glenberg, Gutowski, Lucarelli, and Davidson (2010), in a similar vein, showed that Botox injections affect cognitive processing of emotion-laden sentences through paralysis of facial muscles. Niedenthal (2007) references the ‘reciprocal relationship between the bodily expression of emotion and the way in which emotional information is attended to and interpreted’ (p. 1002). As noted, interventions directing arm movements (e.g., Nathan et al., 2014; Novack, Congdon, Hemani-Lopez, & Goldin-Meadow, 2014) and eye gaze (Thomas & Lleras, 2007) have led to superior performance in mathematics and general problem solving.

ACT theory offers several testable hypotheses about thinking and learning that have implications for STEM education. One hypothesis states that directed actions*,* body movements that learners are instructed to formulate, can induce cognitive states that activate relevant knowledge. A second hypothesis is that action-based interventions by themselves are expected to induce cognitive states around ideas that are not propositionally encoded. In this way, actions can foster insights that may be nonverbal, and therefore unavailable for immediate verbalization. In this manner, action-transduced knowledge may operate outside of the awareness of the learner.

Consistent with these two hypotheses, Nathan et al. (2014) found that experimental participants who performed directed actions that were selected for their relevancy to mathematical tasks showed improved intuition and insight, even though participants were largely unaware of their mathematical relevance or influence. This work raises three important question about the epistemological basis for claims about body-based interventions influencing cognition. The first question is how actions absent any action-based goals can conjure something meaningful. The second question is whether there is evidence that thoughts induced by actions can contain entirely new ideas, or if the conjured ideas are simply due to priming effects of pre-existing knowledge. The third question is how movements performed in response to directions but without inherent meaning can contribute to specific meaning-making.

Several empirical studies speak to this first question, and show that presenting stimuli can activate motor systems even in the absence of any motoric goals to act. Skilled *kanji* writers, for example, demonstrate motor-system activation in areas associated with writing these characters, even without any intention to write (Kato et al., 1999). Isolated word presentation of action words (e.g., pick, lick, kick) can induce motor responses in the associated muscle systems (fingers, tongue, legs; Pulvermüller, 2005). Beilock and Holt (2007) showed that people reported preferences for letter dyads (FJ over FV) without cuing any action-based goals because these were less demanding to type, but this held only for skilled typists. These studies illustrate ways that people invoke action-based meaning for presented stimuli even when action-oriented goals are not explicitly cued.

On the second question, Leung et al. (2012) provide evidence across several experiments that embodied interventions can increase the generation of entirely new ideas, rather than only priming prior knowledge. Here, interventions that embodied creative and alternative viewpoints (changing hands, being outside of a box, freely wandering) led to more creative responses on a number of convergent and divergent thinking tasks.

In addressing the third issue, we note that actions performed in response to directions can generate a specific meaning by evoking many multiple meanings that undergo real-time selection. One way that actions may generate new ideas is through mental simulation (e.g., Barsalou, 2008). Mental simulation processes literally ‘run’ or ‘re-run’ multimodal enactments of external sensory and motoric signals along with internally generated introspective events. This offers one account for why we perceive similarities between enacting, observing, and recalling specific behaviors; and understand the minds and behaviors of others (Decety & Grèzes, 2006). The GAME framework proposed by Nathan and Martinez (2015) provides a computational account of how actions that are initiated without specific meaning contribute to specific meaning-making through mental simulation. The GAME framework builds off the MOSAIC architecture, which provides an account of movement regulation in uncertain environments (Haruno, Wolpert, & Kawato, 2001; Wolpert & Kawato, 1998). In this model, as a movement commences, it simultaneously initiates the parallel production of multiple, paired predictor-controller modules. Each module is intended to anticipate one of the myriad of plausible next states of the motor system. Each predictor-controller pair receives feed-forward signals of expected movement and feedback signals of the actual movement, which provides rapid access to the difference between the projected state of the motor system in the simulated mental model and its actual state as movement occurs. This coupling between actual and simulated motor activity establishes a pathway for transductive influences of actions on the cognitive state of the agent that may start out as nonspecific, and ultimately induce specific, contextually relevant cognitive states. As the movement progresses, there is continuous competition among these predictor-controller modules, each serving as a potential future state of the mental simulation. The system favors those modules that most closely track the external influences from the environment and the internal influences from the current cognitive states. Selection of the most helpful predictor-controller modules is used to update the reader’s current mental simulation, enabling idea generation, while the current action potentially alters current cognitive processes. Those specific predictor-controller pair modules that are found to most accurately predict both the state of the world and the state of mind receive greater activation for the future, thus improving body response and action-cognition alignment. Nathan and Martinez (2015) provide evidence in support of the prediction that the execution of motor control programs during movements such as gesture production can influence simulated mental model construction processes, and enable the generation of novel inferences. In this way, even nonspecific movements can induce specific mental states through ACT that can lead to novel cognitive processing, and support the generation of insights through nonverbal means.

## A GEMC theory of proof-with-insight

Nathan et al. (2014) found that the effect of directed action on producing an informal proof was significantly enhanced when pedagogical hints were introduced to engage participants’ language systems. Without language activation, solvers may experience their insights through nonverbal means, but still be unable to verbally articulate a proof. Consequently, we hypothesize the need for co-activated language and motor systems during task performance for achieving valid proofs-with-insight. It follows from our theory that processes coordinating language and motor systems will, in turn, produce a legacy of semantically rich co-speech gestures, which reveal students’ abstract and generalizable mathematical thinking (Hostetter & Alibali, 2008). GEMC theory posits that dynamic gestures mediate the generation of correct mathematical insight during proof production. Simulated actions that drive performance are specifically evident in students’ generation of dynamic gestures and transformational speech (Pier et al., 2014). Dynamic gestures, to review, are those that manually depict and transform an object. Researchers have identified the importance of dynamic gestures during mental rotation tasks (Göksun, Goldin-Meadow, Newcombe, & Shipley, 2013; Newcombe & Shipley, 2012; Uttal et al., 2012). Our usage of the term aligns best with Garcia and Infante’s (2012) characterization of gestures produced when solving calculus problems, as ‘moving the hands to describe the action that occurs in the problem or movements made to represent mathematical concepts’ (p. 290). Following Harel and Sowder’s (2005) framework, ‘transformational speech’ describes those utterances that indicate (1) logical inferences, where conclusions are drawn from premises; (2) generalization of relevant mathematical relationships between mathematical entities; and (3) operational thought, such that a cohesive argument progresses through a systematic chain of goals. Pier et al. (2014) identified transformational speech patterns as particularly important to valid proofs. Transformational speech was defined as goal-directed manipulations of mathematical objects through conditional statements (‘if … then …’) and language that repeated key mathematical terms as inference was performed. They found that transformational speech and dynamic gesture independently accounted for unique variance in their model predicting proof validity.

**Fig. 2:**
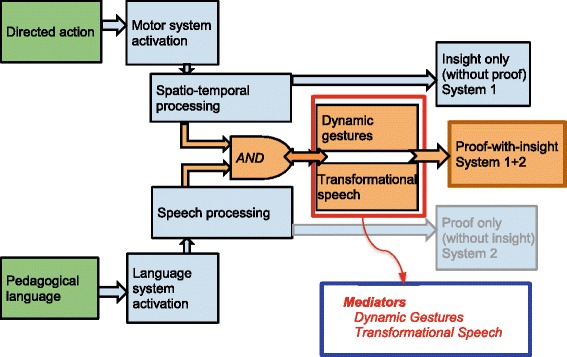
Grounded and embodied mathematical cognition (GEMC) theory. Actions (top pathway) combine with language (bottom pathway) to generate a proof with intuition, which is hypothesized to be mediated by simulated action, as exhibited by the speaker’s dynamic gestures and transformational speech

In our theory (Fig. 2), we show, on the far right, that both dynamic gestures and transformational speech must be coordinated to generate proof-with-insight; that is, to articulate a proof that is mathematically valid, intuitively satisfying, consciously understood by the learner, and available for explanation and reflection (Systems 1 and 2). The theory hypothesizes that activating learners’ action systems without engaging language systems to instill propositional meaning to these actions can lead to the generation of intuitions (System 1) and insights about the conjectures (top pathway of Fig. 2), but that this by itself will not yield a valid proof that presents the chain of analytical reasoning for why the insight holds. Achieving state of insight, while meaningful to the solver, is also not likely to be persuasive to others without an accompanying chain of justification (Harel & Sowder, 2005) This provides an account for why action-based interventions can yield proficiency in automated procedures and perceptual recognition (i.e., engagement of System 1) but fail to support the abstract understanding and reflection that enables verbal explanations, generalization, and far transfer (i.e., engagement of System 2; Evans, 2003).

Prompted speech (e.g., following an authoritative script) can yield the recitation of valid proofs (lower pathway of Fig. 2), but we hypothesize that it does so without generating perceptuo-motor forms of knowing that are characteristic of intuitive understanding (e.g., Kellman & Massey, 2013; Kellman, Massey, & Son, 2010; Koedinger, Corbett, & Perfetti, 2012). Actions and speech production can be independently manipulated to contribute to proof performance, and each may make contributions to students’ mathematics skill and knowledge.

Our central claim is that the coordination of action and language is necessary for students to perform simulated actions of the appropriate mathematical entities. With coordination, students produce dynamic gestures along with concurrent transformational speech that serve as mediators of their mathematical thinking. This enables students to formulate an insightful and explicable chain of reasoning that constitutes a mathematical proof that is both externally valid and internally meaningful.

## Research to practice via learning environment design

One of the great challenges for developing theoretically driven interventions is that learning theories markedly under-constrain the design of instruction and learning environments. In terms of implementation, there are myriad design decisions that will have consequences for learning and engagement that must practically be settled, yet that fall outside of the prescribed learning theory. It is in this sense that some Learning Sciences scholars have argued that learning environment development is more closely aligned with engineering than science (Nathan & Sawyer, 2014) and depends on iterative cycles of learning environment design that inform the specific design decisions as well as the overarching theory.

Based on our theory and the findings reviewed above, we offer novel predictions that guide the design of GEMC-inspired learning environments: directed actions and language prompts are hypothesized to each act as viable, independent, malleable factors for enhancing proof performance by fostering mathematical insight and verbalizable proof production. GEMC theory, along with findings from Nathan et al. (2014), served as the basis for the design of a scalable video game environment that would guide directed actions in service of players’ proofs and justifications for mathematical conjectures. In accordance with GEMC, game play elicited directed actions thought to (unconsciously) foster dynamic gestures that would facilitate insight, while verbal supports were used to provide pedagogical hints that activate language systems and encourage the integration of actions and language. We narrowed our domain of inquiry to focus on high-school planar geometry and expanded the task set within that domain to include multiple geometry conjectures. We recruited age-appropriate participants to investigate how interactive game play that elicited actions and coordinated language could be used to further develop the emerging theory for promoting proof-with-insight and serve as a prototype for a scalable, embodied game for promoting pre-college mathematical reasoning.

### Theoretically motivated hypotheses

**Fig. 3:**
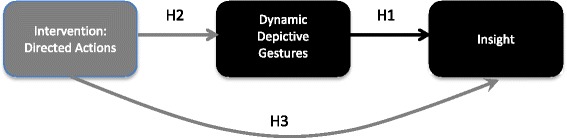
Illustration of hypotheses H1, H2, and H3 for the pilot study. *Black shapes and links* denote predictions about the process account in accord with grounded and embodied mathematical cognition theory. *Gray shapes and links* denote predictions about the intervention designed to improve performance

We offer testable hypotheses about the nature of GEMC. As illustrated in Fig. 3, H1 offers a novel theoretical prediction of a positive association between dynamic depictive gesture production and valid mathematical insights relating to problem tasks. H2 and H3 offer predictions about the influence of directed actions as interventions designed to directly or indirectly influence insight performance. As such, H2 and H3 are a step further from the process account central to GEMC, because implementation choices for when and how to elicit directed actions within the intervention may bear on their influence on outcome measures. H2 predicts that inducing directed actions leads to the production of dynamic gestures. H3 predicts that inducing directed actions improves the generation of mathematical insights. H3 establishes the overall effect that directed actions are a malleable factor for enhancing students’ mathematical insight. Establishing the meditational role of dynamic gestures to produce insights (the H2-H1 path) paves the way for a class of embodied interventions within GEMC to promote STEM reasoning.

- H1. Production of dynamic gestures during proof and justification will predict student performance on mathematical insights.
- H2. Performing mathematically relevant directed actions (without pedagogical language) will predict the production of dynamic gestures.
- H3. Performing mathematically relevant directed actions (without pedagogical language) will predict student performance on mathematical insights.

**Fig. 4:**
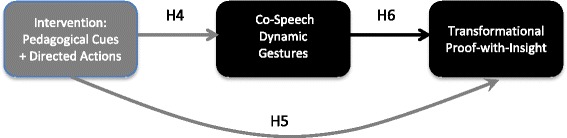
Illustration of hypotheses H4, H5, and H6. *Black shapes and links* denote predictions about the process account in accord with grounded and embodied mathematical cognition theory. *Gray shapes and links* denote predictions about the intervention designed to improve performance

As illustrated in Fig. 4, H4 and H5 examine the influence of interventions that coordinate language with action systems to enhance transformational proof production. H4 predicts that combining pedagogical language prompts with the directed actions (such as those identified in H2) will elicit more dynamic gestures. H5 predicts that pedagogical language prompts combined with directed actions will lead to improved proof performance. H5 establishes the overall effect that mathematically relevant directed actions coupled with language promotes transformational proofs-with-insight, while H4 considers the meditational role of dynamic gestures on proof performance. H6 (as with H1, above) examines the theoretical process account that proof-with-insight performance is associated with dynamic gesture production.

- H4. A language intervention, in the form of pedagogical cues about the mathematical relevance of game-based directed actions, predicts the production of dynamic gestures during proof production that co-occur during transformational speech production.
- H5. An intervention consisting of language cues coordinated with mathematically relevant directed action predicts the performance on production of valid transformational proof-with-insight.
- H6. Co-speech dynamic gestures predict the production of transformational proof-with-insight.

### Pilot study

A pilot study was conducted using a prototype version of a new video game, *The Hidden Village*, designed as a platform to investigate these hypotheses. The pilot study is provided as an initial, illustrative example of how the GEMC framework can be empirically tested and iterated upon. Given the small sample size and early operationalization of the relevant outcome measures, we make only tentative conclusions from these findings, and offer them as a means to refine our ongoing empirical investigation.

#### Methods

**Fig. 5:**
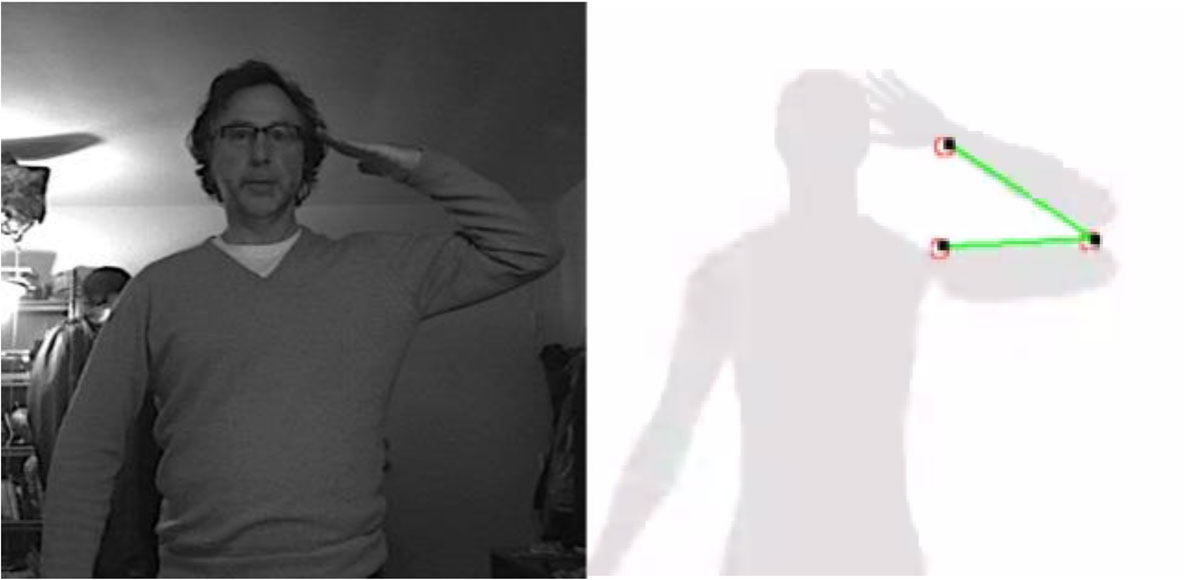
Wireframe skeleton that shows how the software in *The Hidden Village* tracks players’ body movements using images from the laptop camera

**Fig. 6:**
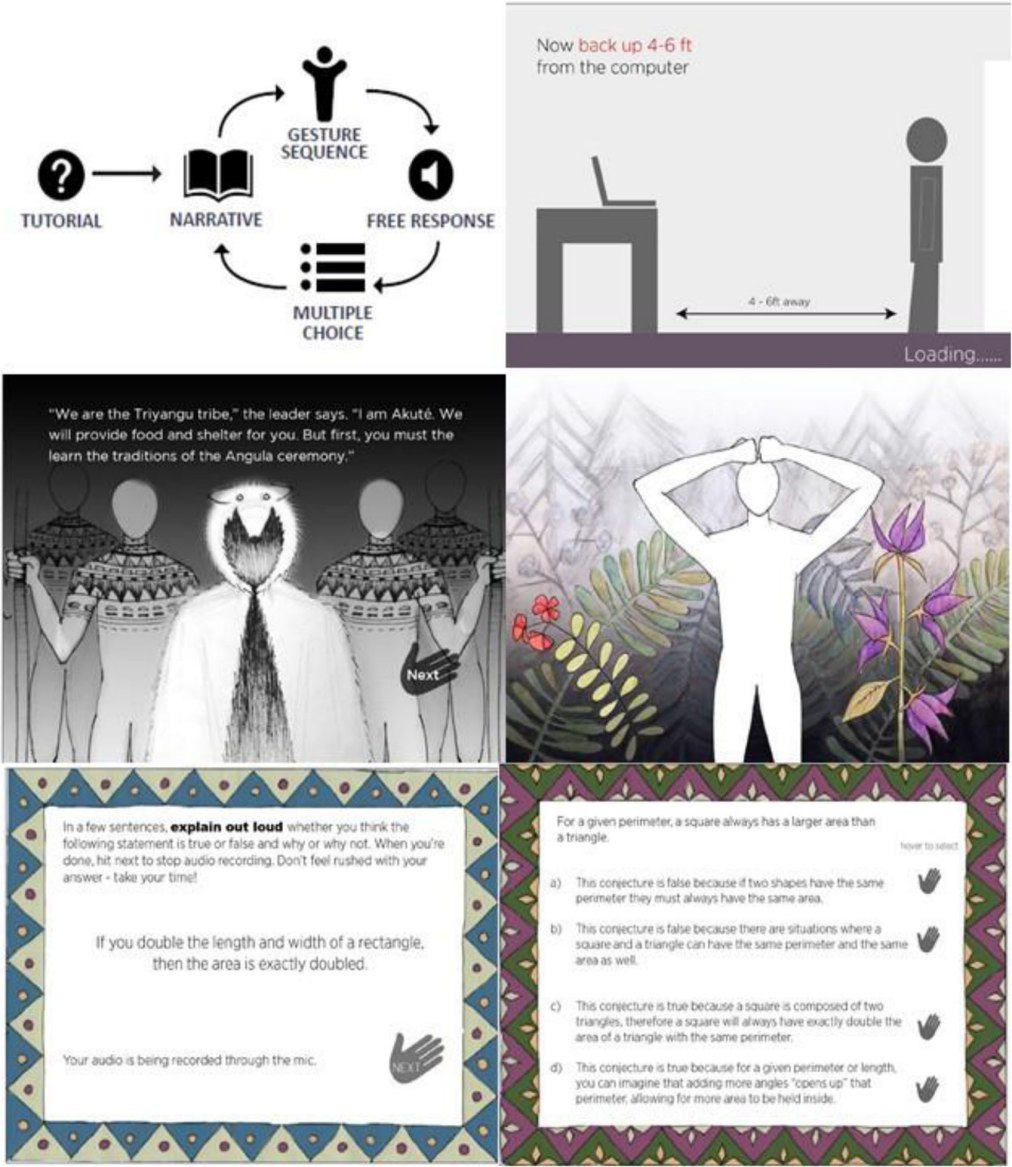
The procedure for playing *The Hidden Village*. The *top left* image shows the flow of the game through five stages, with the circular cycle repeated for each conjecture. The remaining images show screenshots from each of the stages of the game: tutorial (*top right*), storyline (*middle left*), directed action sequence (*middle right*), player’s free response to a geometric conjecture (*bottom left*), player’s multiple choice response to a geometric conjecture (*bottom right*). Multiple choice responses are not considered in the present paper for space

*The Hidden Village* utilizes a standard built-in laptop camera and specialized image processing software developed by Extreme Reality, Ltd. (xtr3d.com) to determine in real time whether the player successfully executes each action sequence. The software generates a wireframe skeleton (invisible to players) to track the coordinates of the user’s joints (Fig. 5) to determine if player actions match the elicited directed actions of in-game avatars. The game playing procedure (depicted in Fig. 6) begins with setup/calibration instructions. The storyline starts when players, lost in the Hidden Village, encounter an imposing tribe whose culture they must accept by copying arm movements at the welcoming ceremony. Movements are designed to either be task-relevant, capturing some key relation of the subsequent geometry conjecture (Table 1), or task-irrelevant. Action relevance is manipulated randomly across conjectures within player. Sequences of movements must be successfully repeated five times for a player to progress through each location in the village. The player is then presented with a geometric conjecture as a challenge from the tribe and instructed to speak aloud with a proof as to why the conjecture is true or false. All speech and actions were recorded during game play.

**Table 1:**
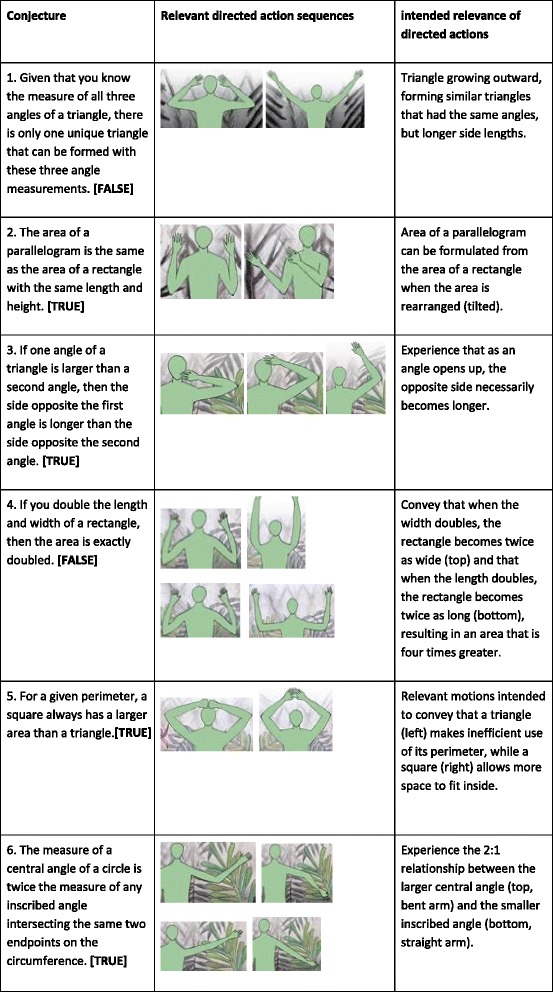
Relevant directed actions and associated conjectures

Irrelevant motions (not pictured) included similar arm movements that did not directly relate to the conjecture.

In the first round of data collection, 18 middle- and high-school students (grades 6 to 11; 16 male, 2 female) attending a video game design summer camp on a university campus individually tested the game; three had previously taken a geometry course. In the second round of data collection, 17 high-school students from the same city (7 male, 10 female) in 9th or 10th grade enrolled in a geometry class at a private school participated in the study. The population at the high school has a mean college admissions American College Testing score of 26, matching the national average.

Students were given a pre-assessment measuring their knowledge of geometric properties of circles, triangles, and quadrilaterals. Students then played the game, experiencing six conjectures in random order. Upon finishing, the interviewer revisited two to four conjectures (depending on time remaining) that the student had received relevant motions for, and revealed that the motions had been relevant by displaying an image that showed the game motions and the conjecture. Participants were asked to think about how their motions might have been connected to the conjecture, and given an additional opportunity to provide a justification following this pedagogical hint.

For scoring student responses, we adopted Harel and Sowder’s (2005) notion of transformational proofs, which are part of a broader category of deductive proof schemes. Harel and Sowder argue that deductive proof schemes constitute ‘the essence of the proving process in mathematics’ (2005, p. 23) and involve operating upon mathematical objects, observing the result, and building upon the proof. As such, transformational proofs have three defining characteristics. First, they are , that is, they show the argument is true for a class of mathematical objects. Second, they involve operational thought, so that the prover progresses through a goal structure, anticipating the results of transformations. Finally, they involve logical inference, such that conclusions are drawn from valid premises. However, as described earlier, we consider such proofs to be ‘informal’ when they are presented verbally to make conceptual sense to a listener, rather than as a mathematically exhaustive logio-deductive written argument.

Videos of students playing the game were divided into segments of each student proving each conjecture, and were transcribed. Students’ responses to each of the six conjectures were scored either 0 or 1 along four dimensions: (1) whether they made any spontaneous depictive gestures while attempting their proof, (2) whether they made any spontaneous dynamic depictive gestures while attempting their proof, (3) whether they recognized the key mathematical insight behind the proof, and (4) whether they formulated a valid informal transformational proof. As noted above, mathematical insights involve an intuitive yet not fully elaborated understanding of the key ideas behind a conjecture. By coding for insight in addition to informal proof, we sought to capture instances where students seemed to have some limited understanding of how the geometric system worked, but were unable to fully formulate and articulate their thinking.

Each of these 0/1 codes was used as a dependent measure in a mixed-effects logistic regression model. This analysis technique was chosen to allow for repeated measures (random effects) to be included for both participant and conjecture. Predictors included gender, pre-test score, highest mathematics course (pre-algebra or lower, algebra, geometry or higher), how many conjectures the participant had proved previously as an ordered factor variable (to account for tiring), and sample (video game camp or private high school). *D*-type effect sizes were calculated using the method in Chinn (2000). In Cohen (1988), effect sizes of 0.2, 0.5, and 0.8 are considered small, medium, and large, respectively.

#### Results

Results of the regression analyses support the trends that are visually apparent from the descriptive statistics in Table 2. When considering H1 (the effect of gesture on insight and proof), producing any depictive gestures (not necessarily dynamic) predicted that participants would formulate the mathematical insight (odds ratio = 3.0, *d* = 0.6, *p* = 0.007), but not formulate an informal proof (*p* = 0.11). However, making dynamic depictive gestures predicted both insight (odds ratio = 8.1, *d* = 1.2, *p* < 0.001) and proof (odds ratio = 11.5, *d* = 1.3, *p* < 0.001). This suggests that producing any depictive gesture may help students glean some key ideas behind the conjecture; however, dynamic gestures may be associated with reasoning about relationships between geometric objects. This supports H1, a novel process claim, which stated that dynamic gestures would predict mathematical insight.

**Table 2:** Descriptive statistics showing average incidence of gesture, insight, and proof for different experimental conditions

Condition	Depictive gestures	Dynamic depictive gestures	Mathematical insight	Transformational proof
Irrelevant actions (*N* = 81)	16.0%	9.9%	28.4%	14.8%
Relevant actions (*N* = 128)	28.1%	10.0%	25.8%	14.1%
Relevant actions + pedagogical language (*N* = 100)	58.0%	29.3%	43.4%	27.0%

*Note*: Standard deviations are not provided because when the outcome is binary (0/1), the sample size and mean give all necessary information

The relevance of the directed actions cued by the video game was manipulated within subjects. Participants were more likely to make depictive gestures on trials for which they had performed relevant (versus irrelevant) directed actions (odds ratio = 4.2, *d* = 0.8, *p* = 0.008). Counter to H2 and H3, in trials where participants performed relevant directed actions they were not more likely to make dynamic gestures (H2), or demonstrate mathematical insight (H3), than in those trials in which they performed irrelevant directed actions (*p-*values > 0.1). Relevant directed actions seemed to activate participants’ motor systems and leave a trace in the form of depictive gestures during proof, but these gestures were often not coded as dynamic gestures. As described above, depictive gestures only predicted insight (not proof), whereas dynamic depictive gestures predicted both insight and informal proof. Note that for these comparisons, most participants (*n* = 27) reported not being consciously aware of the relationship between the directed actions they performed and the conjectures. When participants who did report some conscious awareness were omitted from the analysis (*n* = 8), the pattern of results was the same. In sum, we see the predicted association between dynamic gestures and insight (H1). However, while video game trials cuing mathematically relevant actions were more likely to elicit overall gesture production among players, cuing relevant actions as an in-game intervention did not reliably increase players’ production of the all-important dynamic gestures (H2) or lead to a significant increase in mathematical insights (H3).

As noted, players often had no awareness that the actions they were asked to perform related to the mathematical conjectures. When comparing participant performance before and after the intervention of a pedagogical hint explicitly linking actions to the conjectures (H4 and H5), findings emerged that support GEMC. Following the pedagogical hint, participants were more likely to make depictive gestures (odds ratio = 5.4, *d* = 0.9, *p* < 0.001) and more likely to make dynamic depictive gestures (odds ratio = 4.0, *d* = 0.8, *p* = 0.001), supporting H4 which stated that directed actions combined with pedagogical language would promote dynamic gesture production. In support of H5, when coupling directed actions to pedagogical language, participants were more likely to produce favorable mathematics outcome measures, including expressing the insight (odds ratio = 3.1, *d* = 0.6, p < 0.001) and formulating a valid informal proof (odds ratio = 4.7, *d* = 0.9, *p* < 0.001). In addition, we saw support for our theoretical claim that dynamic gesture production reliably predicts the performance of formulating a valid proof (odds ratio = 5.52, *d* = 0.94, *p* < 0.001), supporting H6. Overall, receiving pedagogical language connecting the directed actions to the conjecture was highly beneficial for players’ mathematics performance, both for insight and for proof production.

Our results suggest that performing relevant directed actions during game play fosters depictive gestures (versus irrelevant actions; *d* = 0.8), and that making depictive gestures predicts mathematical insight (*d* = 0.6). However, surprisingly, performing relevant actions (versus irrelevant actions) did not predict insight, as would be expected. Supplementary analyses showed that the ‘extra’ depictive gestures induced by relevant directed actions tended to be static, non-moving gestures that did not display transformational reasoning. Although trials where participants performed irrelevant actions were less likely to have gestures, responses from irrelevant action trials showed a greater proportion of the all-important dynamic gestures (62.0% of gestures from irrelevant trials versus 36.1% of relevant trials).

#### Qualitative analysis of contrasting cases

To better illustrate students’ reasoning in the context of mathematical proof and directed action, we show two transcripts of how directed actions with pedagogical language influenced participants’ reasoning. These transcripts were selected to illustrate contrasting cases where pedagogical language was effective versus ineffective at allowing students to link their directed motions to a conjecture and to transform the directed motions into their own, personal co-speech dynamic gestures. The transcripts also illustrate how spontaneous gestures, verbal reasoning processes, pedagogical language, and the directed motions all come together to allow for successful or unsuccessful proof attempts.

**Fig. 7:**
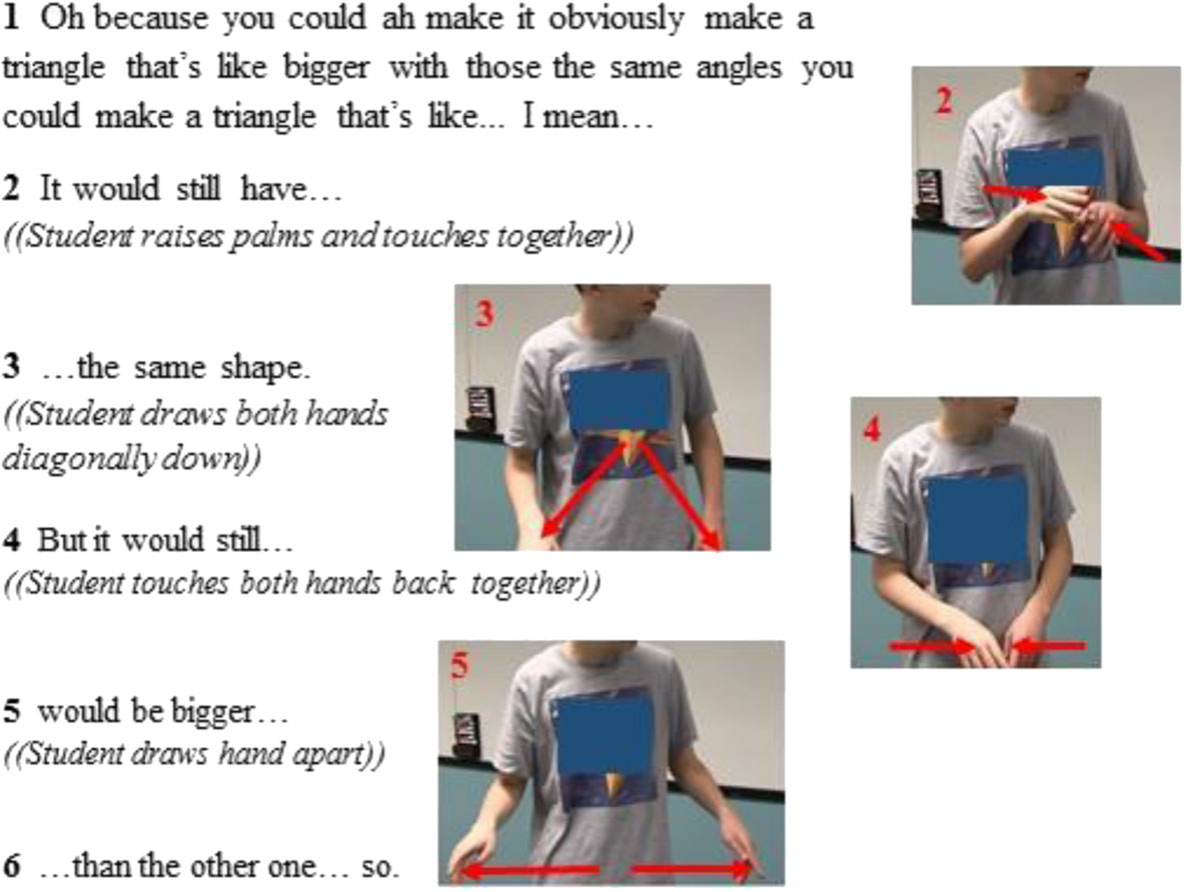
Photo transcript of student proving Conjecture 1 in Table 1 after receiving a hint that his directed actions are relevant to the conjecture. *Red lines* show motion and inferred shapes

Figure 7 shows a photo transcript of a student formulating a new proof for the conjecture that only one unique triangle can be formed from three angle measurements (Conjecture 1 in Table 1) after receiving a hint that his directed actions had been relevant. Initially, the student had incorrectly said the conjecture was true because angle measurements are unique to a triangle. After the hint, his verbal proof showed a growing triangle as a means of disproving the conjecture. To make his argument, he utilized co-speech spontaneous dynamic gestures of a triangle growing outwards, indicating the directed motions may have left a legacy (Donovan et al., 2014).

**Fig. 8:**
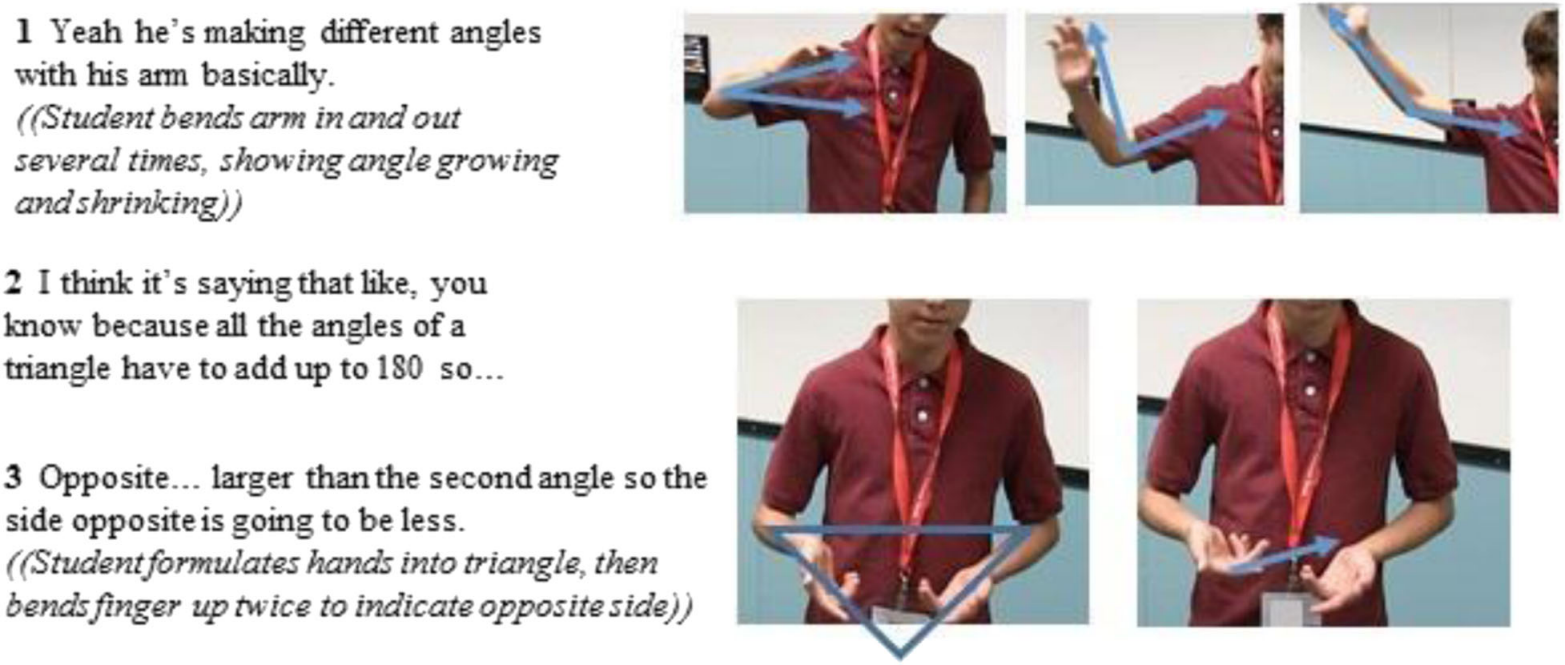
Photo transcript of student providing incorrect proof to Conjecture 3 in Table 1 after receiving a hint that his directed motions are relevant to the conjecture. *Blue lines* show motion and inferred shapes

However, relevant directed actions with pedagogical language were not always effective for students. Figure 8 shows a contrasting case where a student’s reflection upon the directed motions was unsuccessful for Conjecture 3 in Table 1 (side opposite largest angle is largest). He seems to catch on to the intended insight that the angle in the triangle was growing, and imitated the dynamic arm movement he had been asked to perform (Line 1). Later, when he formulated his proof, he did not use the intended relation, however. He maintained that the conjecture was false because angles needed to add up to 180° (Line 2) and made a non-dynamic depictive gesture that inaccurately indicated a large angle corresponding to a smaller side length (Line 3). This may be an example of an unhelpful insight garnered by a strong association between triangles and angles adding up to 180°.

#### Discussion

Our findings from the pilot data are interesting, to say the least. And while our pilot sample sizes are modest, and there are reasons to question whether the two student samples can be combined, the combined results provide valuable early information that can be used to inform our prototype intervention and the theory.

The model of insight performance shown in Fig. 3 explores important theoretical and practical claims for advancing GEMC in an area of advanced mathematics. In support of H1, dynamic gestures led to improvements in insight, which is a novel contribution to theories of embodied cognition for promoting STEM. However, findings contrary to H2 show that is an understanding of how to design the interventions that induce those dynamic gestures that is lacking. Although the directed action cues did significantly influence students’ gesture production, most of the gestures were not dynamic and did little to support students’ transformational proof production. The increased production of gestures overall following game play trials with relevant directed actions is itself a curiosity given that, as we noted, most players had no idea these directed actions were related to the conjectures. This suggests that the intervention is influencing unconscious reasoning associated with System 1 processes.

H3 incorrectly predicted that relevant directed actions elicited without pedagogical hints that signaled their mathematics relevance would promote insight. H3 makes a claim that bypasses the hypothesized mediational role of dynamic gestures in shaping mathematical reasoning. Thus, at this preliminary stage, we were not able to identify a simple way to improve insight solely by manipulating the relevance of the directed actions within an intervention. A *post hoc* analysis did show, to our surprise, that irrelevant action cues were actually more efficient at eliciting dynamic gestures than were task-relevant cues, in that a higher proportion of the total gestures that were produced were dynamic. In essence, students who received relevant directed actions were gesturing more, but this improvement in gesture rate was not impacting the production of the more influential dynamic gestures. Indeed, for both the relevant and irrelevant conditions, the overall tendency to produce dynamic gestures on the first trial was exactly the same - around 10% of trials showed dynamic gestures (see Table 2). These findings bring to light the chasm between learning theories and their application by highlighting ways that design decisions for the intervention are under-constrained: we see academic benefits when students produce dynamic gestures, but we do not yet know how best to elicit them.

The findings concerning the effects of explicit language connecting the actions to the conjectures also provide important information for the emerging theory and game design. Consistent with the predictions made by H4 and H5, players were significantly more likely to make dynamic gestures (H4) and more likely to generate correct insights and valid informal proofs (H5) after receiving verbal hints. This provides tentative support for the GEMC theory and its call for coordinating language and action systems in service of mathematical reasoning. It raises a question of why the pedagogical hints improved insights, which we take as nonverbal. One indicator is that pedagogical hints were found to influence insight performance directly, and to increase the production of dynamic gestures, which, as hypothesized earlier (H1), promotes mathematical insight and the formulation of valid informal proofs (H6) through a mechanism such as transduction. As predicted, we also found support for H5, the applied path that bypasses the meditational influence of dynamic gestures and predicted a direct relationship between pedagogical language coordinated with directed action and our primary outcome measure of interest, proof-with-insight. Although these results need to be replicated and generalized across a broader set of mathematical conjectures and student populations, these initial findings are encouraging for future game designs that instantiate this direct pathway.

A qualitative analysis of contrasting cases highlighted the significant challenges involved in transforming a directed motion into a meaningful dynamic gesture that makes sense to the learner in the context of the task. This transformation has the potential to support students’ proving activities and give them powerful new body-based resources with which to confront the task, as shown in the first transcript. However, making the mapping between directed actions and the sequence of key mathematical ideas needed to prove a conjecture is fraught with difficulty, as shown in the second transcript. This reveals a central challenge in the use of directed actions as an embodied intervention for learning: how to select and design the actions such that the mapping is as sensible and accessible to the learner as possible.

### Summary of findings

The current study provides empirical support for an emerging theory of cognition-action transduction for embodied mathematical cognition. The production of dynamic depictive gestures is strongly predictive of mathematical insight (H1) and informal mathematical proofs (H6), providing support for the GEMC process account. While game-initiated directed actions increased depictive gesture production, this did not translate to improved mathematical reasoning because these depictive gestures were often not dynamic (H2), suggesting that the directed motions utilized by the intervention may need further development.

This highlights the challenges of designing theory-based learning technologies, and reinforces the importance of iterative design approaches for effective educational games.

Our findings also support the hypothesis that pedagogical language directing students to the relevance of the game-directed actions improves both dynamic gesture production (H4) and proof performance (H5), which suggests that the language aspects of the intervention were effective. This supports the view that integrating language systems with nonverbal motor-based forms of knowledge can enhance analytic forms of mathematical reasoning.

## Conclusions

This work offers several contributions, both theoretical and practical, for promoting mathematics education. By focusing on geometry and proof, we contribute to an area of mathematics that makes substantial interconnections across the STEM fields, including classic fields such as physics, chemistry, and biology, as well as more recent advancements, such as nanotechnology and fractals.

From a theoretical perspective we have begun to articulate and investigate a theory of embodied cognition. Our theory makes an overt distinction between insight, or System 1 thinking, as a nonverbal, unconscious form of knowing of mathematical relationships, and conscious, analytic formulation of propositional knowledge, or System 2 thinking, that supports the production of logically valid transformational proofs. While action systems and language systems each contribute to both insight and proof, proof-with-insight, it is hypothesized, depends on a coordination of these two systems. On these matters, we showed partial success. However, the shortcomings offer up valuable direction for future models and research.

On a practical level, our work contributes to issues concerning the design of theory-motivated interventions for improving STEM education, and the proliferation of commercial body-based programs in STEM and language education. One important take-away is that GEMC is not about thoughtless movement. Rather GEMC considers the integration of nonverbal and verbal forms of (mathematical) thinking.

### Deriving design principles from theory and research

Lee (2015) observes that as new theoretical perspectives, like embodied cognition, emerge in education, so do new ways of using technology to support the difficult tasks of teaching and learning. Technology can support the construction of mathematical meanings (National Council of Teachers of Mathematics, 2000), allowing students to ‘play with’ mathematics (Fey, 1989) and explore justification and proof in a visual, interactive environment (Hanna, 2000). Technology also offers novel opportunities for the embodiment of mathematical ideas (Lee, 2015), allowing students to enact mathematical activities related to visualization, symbolization, intuition, and reasoning. We are witnessing a new genre of educational technologies and interventions for promoting STEM, routed in theories of embodied cognition.

GEMC offers theoretical guidance for the design of effective learning environments. Working from this paradigm, Lindgren and Johnson-Glenberg (2013) offer six ‘precepts’ for the design of embodied and mixed-reality learning environments. Their recommendations emphasize ‘gestural congruency,’ where learners’ actions are structurally or analogically related to the symbols and their meaning” (p. 446). From this general recommendation, we draw out two design principles for promoting mathematical proof: (1) eliciting dynamic depictive gestures through relevant directed actions that are congruent with the geometric relations that are present in the conjecture tasks; and (2) activating language systems that are explicitly coordinated with the relevant action systems, through pedagogical cues. *The Hidden Village* served as an intervention medium for these design principles, and attained partial success on the impact of language cuing. We were also able to add further empirical support for the theoretically inferred role of dynamic gestures on insight. However, we were not successful at effectively eliciting dynamic gestures on demand through elicitation of relevant directed actions by copying the motions of in-game characters.

One of the key design challenges when creating an environment that directs motions for a sophisticated conceptual area like geometric proof is determining which actions to direct to best facilitate students’ reasoning. One idea is to examine the gestures, particularly the dynamic gestures, that competent problem-solvers tend to make, and then turn these gestures into directed actions given by the game for particular conjectures. However, this is complicated by limitations in current technology for accurately detecting certain types of motions. While subtle hand gestures are more difficult for our prototype video game to identify, large arm movements are less problematic. Thus our general approach is to examine the gestures that competent problem-solvers make when they prove our geometric conjectures, and then re-imagine these gestures as large arm movements that the technology can reliably capture. However, some of our motions designed in this way certainly worked better than others.

One finding in the intervention used in Nathan et al. (2014) that has particular relevance was that students who performed directed actions for the Gear task were more likely to have the mathematical insight, but when explicitly prompted to connect their directed actions to the conjecture through pedagogical language, their reasoning and proof performance actually declined. This is because when subject to explicit inspection, the directed actions did not make sense to participants in the manner in which they were intended. Participants would try to imagine how the directed actions were intended to embody the problem solution, and would in some cases guess incorrectly and be led down the wrong path as a result. The same thing occurred occasionally in the pilot study of the video game. For example, some participants who made the two sets of doubling motions for the false conjecture about how doubling the length and width of a rectangle doubles the area actually considered the sets of motions separately, and gave an incorrect proof relating to doubling rather than quadrupling. This is an interesting design challenge - the very motions that promote insight may be problematic when subjected to explicit reflection in order to promote proof. Perhaps prompting players to think about certain unconscious actions and perceptions instills a ‘verbal overshadowing effect’ (Schooler, Ohlsson, & Brooks, 1993), where explicit attention interrupts critical, nonverbal processes involved in insight formation by attempting to propositionalize and verbalize them. Directed motions and pedagogical prompts need to be carefully selected such that they promote both insight and proof, without making learners overly attentive to inappropriate aspects of the task domains.

Pedagogical language connecting relevant motions to the mathematical task at hand are clearly critical to the success of such an embodied learning environment to promote valid reasoning, but little is known about how the pedagogical prompts should be delivered. Nathan et al. (2014) explored the timing of verbal cues and found that prompts delivered before students had the opportunity to prove the conjecture were far less effective than hints delivered after one attempt at proof had already been made. The timing of the pedagogical cues appears to be an important consideration.

The challenges of deriving effective designs to promote learning is, in part, a limitation of the scientific method as a means to translate theory to practice. An analogy to the physical world makes this plain. Newton’s second law (*F* = *ma*) can help analyze why a bridge stands or falls, but does little to inform bridge design. That is the purview of engineering; for it is within the carefully monitored process of the iterative design cycle that one can rapidly explore, test, and converge on successful designs. Research methods such as design-based research (e.g., Barab & Squire, 2004) offer methodological guidance for the process of applying evidence-based research to the design of learning environments. It is within this space we intend to continue to explore how best to elicit dynamic gestures, and harness their cognitive potential for mathematical insight.

Our future research is informed by the findings and limitations of the current study. One immediate action item is to replicate our findings with a larger, more uniform sample of participants from an age/grade-appropriate context. To this end, we will conduct classroom studies with *N* = 150 high-school geometry students across two sites to investigate how directed action and pedagogical language influence insight and proof validity. The findings regarding the lack of an effect of task-relevant directed actions on dynamic gesture production raise questions of the selection of directed actions for instilling dynamic gestures. Two competing hypotheses arise here. One is that our understanding of the principle of gestural congruency, that is, structurally matching learners’ directed actions to geometric concepts, is inadequate in this domain, and we need to develop a more valid analytic process to identify the underlying mathematical ideas for each conjecture and translate them to ‘congruent’ directed actions. Another hypothesis is that we have chosen the appropriate task-relevant directed actions, but have overworked the motor system for these specific actions (by making players match the actions five times before progressing) and thereby inhibited the associated conceptual and spatial relations. Irrelevant actions would, by design, be unrelated to the geometric relations, and therefore may be easier to ignore when learners are confronted with a statement to prove using their already-taxed motor system. Our remedy here is to vary the number of times players have to match the directed actions and see how this impacts insight performance.

Our preliminary results suggest that directed actions in combination with pedagogical language promoted valid informal proofs, and that dynamic gestures played a key, mediating role. While pedagogical prompting yielded some of the strongest findings in support of GEMC, we still understand very little of how these cues foster valid proof production. In future studies we will vary the kinds of pedagogical cues, self-cueing, and the timing of cues in order to understand the robustness of this for promoting mathematical reasoning. We also plan to look at the impact of providing language input alone, without directed actions, on proof performance. Finally, our near-term interest is to introduce this video game to high-school geometry classrooms to support collaborative proof production and collaborative authoring of new mathematical conjectures to provide a generative learning environment for exploring mathematical reasoning about objects and space.

### Commercial programs for embodied learning

One final point addresses the proliferation of motion- and body-based interventions for promoting mathematics learning. There is currently no definitive compendium of these programs, or a systematic inventory of their claims. But programs such as The Action Based Learning™ Lab, MATHS DANCE, and Math in Your Feet generally share intertwined goals of incorporating movement into mathematics instruction for intellectual, physical education and health, and interest purposes. Most of these programs lack the research reporting that would support the claims made about advancing academic goals. However, they are tapping into a growing awareness of the role the body plays in learning and engagement. As our current work matures, we hope to provide sound, empirically tested, and theoretically motivated design guidelines for programs that use embodiment to advance STEM learning. With such a vast design space before us, scholars working in embodied cognition for education will find value and inspiration in programs developed by mathematics educators, dancers, biomedical engineers, and mathematicians, as well as those designed by psychologists and educational researchers. Theoretical advancements in GEMC have the potential to provide a common framework for future efforts to design embodied learning experiences to enhance STEM education.

## Abbreviations

ACT: Action-cognition transduction
GEMC: Grounded and embodied mathematical cognition
STEM: Science, technology, engineering, and mathematics
